# Genomic sovereignty for health equity in Latin America and the Caribbean

**DOI:** 10.1016/j.lana.2026.101490

**Published:** 2026-05-05

**Authors:** Gabriel da Luz Wallau, Túlio de Lima Campos

**Affiliations:** a Núcleo de Bioinformática, Instituto Aggeu Magalhães, Fundação Oswaldo Cruz (Fiocruz Pernambuco), Recife, Brazil; b Departamento de Entomologia, Instituto Aggeu Magalhães, Fundação Oswaldo Cruz (Fiocruz Pernambuco), Recife, Brazil; c Universidade Federal de Santa Maria, Santa Maria, Brazil; d Department of Arbovirology and Entomology, Bernhard Nocht Institute for Tropical Medicine, Hamburg, Germany

Negotiations on pathogen access and benefit sharing, including those linked to the WHO Pandemic Agreement, are reshaping how countries think about genomic data.^1^ For governments in developing regions, genomes have become strategic public health assets tied to industrial policy, diplomacy, and sovereignty.^2^ Rapid sequence sharing now underpins global genomic surveillance strategies for pathogens with pandemic and epidemic potential.^3^ Yet many Latin American and Caribbean countries contributed samples, data, and trial participants while facing delays in vaccine access, limited local manufacturing, and little influence over the rules that govern data use and intellectual property.^4^ The central question for the region remains: under what conditions sharing genomic data generates tangible, reciprocal health benefits, rather than reproducing extractive patterns under the banner of open science?

We argue that genomic governance in developing countries of the Americas must move beyond a narrow focus on data sharing towards a model that links openness to benefit sharing, regional capacity building, and co-ownership of technologies. Human and pathogen genomics now sit at the intersection of public health, industrial development, and international relations. Treating genomic information purely as a global public good, without acknowledging power asymmetries, risks entrenching dependence rather than advancing equity.^5^ Therefore, a regional agenda for genomic sovereignty is needed so that Latin American and Caribbean countries participate as equal partners in setting priorities, building infrastructure, and reaping the benefits of innovation.

Pathogen genomics has become indispensable for outbreak detection, vaccine strain selection, and antimicrobial-resistance surveillance.^6^^,^^7^ During COVID-19, rapid open sharing of viral sequences allowed researchers to track variants and accelerate vaccine development, but governance of those data did not guarantee equitable access to vaccines, diagnostics, or therapeutics.^8^ Many countries in the region expanded sequencing capacity under emergency funding but still lack long-term resources for bioinformatics, data storage, and maintenance of high-throughput platforms. Sequence data generated in Latin America and the Caribbean are often deposited and analysed using infrastructures controlled elsewhere, reinforcing the separation between where epidemiological intelligence originates and is most needed and where it is turned into products.

In the context of human genomics, the persistent under-representation of Latin American and Caribbean ancestry in global databases is a direct driver of clinical risk and healthcare inequality.^9^ Variants classified as pathogenic in largely European and North American datasets may be benign in other populations, leading to overdiagnosis, underdiagnosis, or misdirected interventions when interpretation tools are applied uncritically to highly admixed populations.^10^ Precision medicine that relies on non-representative reference panels risks being least precise where health systems are already most fragile. Addressing these gaps requires sustained investment in region-specific genomic reference data, local infrastructure and capacity for analysis and interpretation, so that genomes are interpreted through frameworks reflecting local ancestries and disease burdens.

These dynamics have sharpened interest in “genomic sovereignty”, as governments recognise that human and pathogen sequence data are strategic resources linked to national security, industrial development, and ultimately power in global health diplomacy.^1^^,^^2^ Over-restrictive data policies could fragment surveillance and slow collective responses, but unrestricted one-way data flows from low- and middle-income countries to high-income analytics hubs risk reproducing a division of labour in which some countries provide raw data while others control platforms, intellectual property, and supply chains.^5^ The challenge for Latin America and the Caribbean is to maintain rapid, cooperative data sharing while embedding benefit-sharing mechanisms and regional capacity building into the architecture of genomic research and surveillance.

A more equitable genomic order in the Americas will require treating genomic governance as part of broader regional integration and industrial policy.^2^^,^^3^ Regional blocs like CELAC, Mercosur, CARICOM; and health agencies such as PAHO and CARPHA can help articulate shared positions that tie data sharing to concrete expectations: access to countermeasures, regional manufacturing capacity, co-development of platforms, and meaningful participation in setting research agendas. National governments should recognise sequencing, bioinformatics, and secure data infrastructures as essential public health components, not optional research luxuries. Global partners, including industry, must accept that ethical engagement with genomic data from the Americas entails clear commitments on technology transfer, co-manufacturing, and transparent intellectual-property terms.^1^

For Latin America, the Caribbean, and the wider Global South, the genomic era will be judged not only by how much data is shared, but by whether sharing genomes also means sharing power, infrastructure, and life-saving technologies. A fair genomic order would treat genomic information as both a global public good and a strategic public health asset, embedding obligations of redistribution and capacity building so that the region moves from being primarily a supplier of samples and data to being a co-designer and co-owner of genomic knowledge and products derived from it.

## Contributors

GLW and TLC contributed equally to the conception, writing and reviewing of the manuscript.

## Disclosure of AI or AI-assisted technologies

The authors used the large language model Perplexity, powered by GPT-5.1 (OpenAI), during manuscript preparation to assist with language editing, condensation of text to meet word-count requirements, and rephrasing of existing arguments for clarity. All AI-assisted outputs were critically reviewed, verified, and extensively edited by the authors, who accept full responsibility for the integrity and accuracy of the work.

## Declaration of interests

The authors declare no conflicting interests.
